# National trends in emergency room diagnosis of pulmonary embolism, 2001–2010: a cross-sectional study

**DOI:** 10.1186/s12931-015-0203-9

**Published:** 2015-03-24

**Authors:** Andrew J Schissler, Anna Rozenshtein, Neil W Schluger, Andrew J Einstein

**Affiliations:** Department of Medicine, Columbia University Medical Center, New York, USA; Department of Radiology, Columbia University Medical Center, New York, USA; Department of Medicine, Division of Pulmonary, Allergy, and Critical Care Medicine, Columbia University Medical Center, New York, USA; Department of Medicine, Cardiology Division, Columbia University Medical Center, New York, USA

**Keywords:** Pulmonary embolism, Emergency department, Computed Tomography (CT) pulmonary angiography

## Abstract

**Background:**

Little is known about the United States diagnosis and burden of pulmonary embolism (PE) in the emergency department (ED), and their evolution over the past decade. We examined nationally representative data to evaluate factors associated with and trends in ED diagnosis of PE.

**Methods:**

We conducted a cross-sectional study using National Hospital Ambulatory Medical Care Survey (NHAMCS) data from January 1, 2001 to December 31, 2010. We identified all ED patient visits where PE was diagnosed and corresponding demographic, hemodynamic, testing and disposition data. Analyses were performed using descriptive statistics and multivariable logistic regression.

**Results:**

During the study period 988,000 weighted patient visits with diagnosis of PE were identified. Among patients with an ED visit, the likelihood of having a diagnosis of PE per year increased significantly from 2001 to 2010 (odds ratio [OR] 1.091, 95% confidence interval [CI] 1.034-1.152, P = 0.002 for trend) when adjusted for demographic and hospital information. In contrast, when further adjusted for the use of computed tomography (CT) among patients in the ED, the likelihood of having a diagnosis of PE per year did not change (OR 1.041, 95% CI 0.987-1.097, P = 0.14). Overall, 75.1% of patients seen with a diagnosis of PE were hemodynamically stable; 86% were admitted with an in-hospital death rate under 3%.

**Conclusions:**

The proportion of ED visits with a diagnosis of PE increased significantly from 2001 to 2010 and this rise can be attributed in large part to the increased availability and use of CT. Most of these patients were admitted with low in-hospital mortality.

## Background

Pulmonary embolism (PE) is a major national health problem representing the third most common cause of death from cardiovascular disease, following heart attack and stroke [1,2]. The estimated annual economic burden of PE in the United States (US) exceeds $8.5 billion [3]. Prompt diagnosis and treatment with anticoagulation has been shown to significantly improve survival [4]. Over the past decade, multidetector-row computed tomography pulmonary angiography (CTPA) has become the primary tool for the detection of PE [5]. CTPA takes minutes to perform and is readily available in most hospital emergency departments (EDs). It is therefore not surprising that a significant proportion of PEs are diagnosed and treatment initiated while the patient is still in the ED [6].

Recent studies have demonstrated a rise in PE incidence over the past decade commensurate to the increased availability and use of CTPA [7-9]. There is growing evidence that we may be diagnosing a different, less severe spectrum of disease with widespread CTPA utilization [7-9]. At the same time, despite data demonstrating that approximately half of PEs are diagnosed in the emergency setting [6,10], few studies have focused on the epidemiology of PE in the ED. One 22-center study recently reviewed the clinical characteristics and treatments used for suspected or confirmed PEs, but it is difficult to extrapolate national estimates from these data [10]. No prior study, to our knowledge, has assessed US trends in and factors associated with ED visits with diagnosis of PE at the national level since the introduction of CTPA.

This study was designed to assess the burden of PE diagnosed in US EDs since the widespread availability of CTPA. The National Hospital Ambulatory Medical Care Survey (NHAMCS) collects objective and reliable information about services provided in EDs in the US. Using this nationally representative sample from 2001 to 2010, we reviewed patient demographic data, signs and symptoms, diagnostic studies ordered and treatments administered in the ED. We further assessed patient disposition (e.g. admission to the hospital), and when possible outcome data (e.g. death during hospitalization). We then evaluated for the presence of temporal trends in the burden of disease.

## Methods

### Data source

The NHAMCS is a sample survey of hospital-based outpatient and ED settings conducted by the Ambulatory and Hospital Care Statistics Branch of the National Center for Health Statistics (NCHS), Centers for Disease Control and Prevention (CDC). The survey is designed to be nationally representative of the utilization of hospital ambulatory medical care services. It uses a 4-stage sampling design that narrows from geographic sampling units to hospitals within these areas to emergency service areas within these hospitals to specific patient visits. Each individual patient visit is weighted using the product of the corresponding sampling fractions at each stage in the sample design to produce national estimates. The NCHS then adjusts sampling weights for survey nonresponse. A detailed description of the survey sample design and data acquisition methods can be found on the CDC website [11]. The NHAMCS is approved annually by the Ethics Review Board of NCHS with a waiver of the requirement to obtain informed consent.

### Study sample

The study period January 1, 2001 to December 31, 2010 was selected to represent the burden of PE after the introduction of CTPA in 1998 [7]. NHAMCS data from 1998–2000 does not contain hemodynamic data. Moreover, it may represent a period when many EDs did not yet have CTPA and therefore these data were not included. NHAMCS data from after 2010 is not yet publicly available for review.

The NHAMCS records up to three diagnoses for each visit, which are later converted to International Classification of Disease, Ninth Revision (ICD-9) codes. The ICD-9 codes used to extract all visits with a diagnosis of PE were 415.11 and 415.19. These codes have previously been validated in administrative data [12]. Sensitivity analysis was performed excluding visits where PE was listed as the non-first diagnosis. ED visits were stratified by patient demographics and expected primary source of payment (private/commercial insurance, Medicare/Medicaid, and other). We subcategorized age into those patients under 65 years-old and those 65 years and older, the age cutoff used in the revised Geneva score [13]. We examined clinical characteristics including vital signs at presentation (heart rate [HR] and systolic blood pressure [SBP]), and use of diagnostic testing (computed tomography [CT] and/or magnetic resonance imaging [MRI]). We defined hemodynamically stable patients as those having HR < 110 beats per minute and SBP > 100 mmHg, based on cutoffs from the pulmonary embolism severity index for prognostication in patients with acute symptomatic PE [14,15]. We categorized disposition into admission (NHAMCS value of admitted to the hospital, admitted to ICU/CCU, admitted to observation, transferred to other medical facility), death (NHAMCS value of death on arrival or death in ED), and all other dispositions (e.g. discharged or left against medical advice). Finally, we collected limited information about the hospitals including geographic region (Northeast, Midwest, South, and West) and whether the hospital was part of a Metropolitan Statistical Area (MSA). US population data were obtained using US Census Bureau intercensal resident population estimates [16]. A small proportion (<10%) of variables had missing data and these visits were excluded from the relevant analyses.

NHAMCS collected a combined CT and MRI variable between 2001 and 2004. We combined MRI and CT variables from the subsequent years to generate a dichotomous variable throughout the study period that indicates if a CT and/or MRI were performed during the patient visit. We then determined the percentage of patients who underwent MRI alone from 2005–2010 to assess the impact of combining the MRI and CT variables.

### Statistical analysis

Descriptive statistics were determined for patient visit characteristics and multivariable logistic regression controlling for patient demographics (age, race, sex) and hospital information (region, Metropolitan Statistical Area status) was used to assess any trends in PE diagnoses among patients who had ED visits over time. We performed an additional multivariable logistic regression controlling for CT and/or MRI use in addition to patient demographics and hospital information. The NCHS guidelines state that estimates based upon fewer than 30 raw observations or with a relative standard error greater than 30% are unreliable. To ensure greater than 30 raw observations, we stratified visits into 2 year blocks for national estimates and figures. We also combined the first 5 years of the study period (2001–5) and compared these data from the subsequent 5 years (2006–10) to assess any for any changes. This ensured most variables contained sufficient raw data to generate robust national estimates. All statistical analyses were performed in Stata/SE 11.0 and 13.0 (StataCorp, College Station, Texas) using the *svy* command to account for the NHAMCS sampling methodology [17].

## Results

A total of 357,681 patient visits representing 1,182,758,000 weighted ED visits (95% confidence interval [CI] 1,060,000,000-1,300,000,000) within NHAMCS from 2001 to 2010 were reviewed. Of these, 283 ED visits representing 988,000 weighted visits (95% CI 783,000-1,193,000) were identified with diagnosis of PE (approximately 0.08% of total weighted ED visits). Among these, 64.0% (95% CI 60.6-67.5%) had PE as the first listed diagnosis. Sensitivity analysis excluding visits where PE was not listed as the first diagnosis showed no significant change in our results.

Table 1 includes a summary of demographic, clinical and hospital information for visits with a diagnosis of PE and all other ED visits for comparison. The mean age of patients seen in the ED with a diagnosis of PE was 59 years-old as compared to a mean age of 36 years-old in patients seen in the ED for all other reasons (p < 0.0001). A greater proportion of patients with a diagnosis of PE were white (80.1%) when compared to patients in the ED for all other diagnoses (73.8%), (P = 0.022). There were also regional differences, with a greater proportion of visits for PE in the Midwest and West (P = 0.021). Most visits to the ED were in Metropolitan Statistical Areas for both patients with a diagnosis of PE and all other ED visits (P = 0.57). There was no statistically significant difference in sex between ED visits with a diagnosis of PE and all other ED visits with women representing a greater proportion (approximately 54%) in both groups (P = 0.88).

**Table 1:** **Demographic and clinical data**

		**ED visits for PE**			**All other ED visits**		
**Characteristic**		**No. of obs.**	**Weighted %**	**(±95%** **CI)**	**Weighted %**	**(±95%** **CI)**	**P-value**
Overall		283	0.08%	0.01%	99.92%	(0.77%)	
Sex	Male	123	45.2%	(3.8%)	45.8%	(0.7%)	0.88
	Female	160	54.8%	(3.8%)	54.2%	(0.7%)	
Age category	Under 65 years	162	55.8%	(3.4%)	85.2%	(0.2%)	<0.0001
	65 years and over	121	44.2%	(3.4%)	14.8%	(0.2%)	
Race/ethnicity	White	223	80.1%	(3.2%)	73.8%	(1.1%)	0.022
	Black	55	19.1%	(3.2%)	22.7%	(1.1%)	
	Other	5	0.8%	**	3.5%	(0.3%)	
Payment source	Private insurance	104	36.4%	(3.3%)	37.0%	(0.5%)	<0.0001
	Medicare/Medicaid	148	55.6%	(3.1%)	41.6%	(0.5%)	
	Other	26	8.0%	**	21.5%	(0.4%)	
Region	Northeast	55	17.4%	(3.4%)	19.0%	(1.7%)	0.021
	Midwest	85	29.9%	(4.6%)	22.9%	(2.4%)	
	South	81	29.0%	(5.3%)	39.8%	(2.7%)	
	West	62	23.8%	(4.6%)	18.3%	(1.9%)	
Hemodynamics	Unstable	71	24.9%	(3.5%)	24.7%	(0.3%)	0.96
	Stable	203	75.1%	(3.5%)	75.3%	(0.3%)	
CT or MRI	Yes	154	57.7%	(4.0%)	11.6%	(0.3%)	<0.0001
	No	129	42.3%	(4.0%)	88.4%	(0.3%)	
Disposition	Admission/Transfer	233	86.3%	(2.7%)	14.7%	(0.3%)	<0.0001
	Other	43	10.4%	(2.0%)	85.1%	(0.3%)	
	Death on Arrival/in ED	7	3.4%	**	0.2%	(0.01%)	
MSA	MSA	243	85.5%	(4.5%)	83.4%	(3.3%)	0.57
	Non-MSA	40	14.5%	(4.5%)	16.6%	(3.3%)	

**Indicates insufficient observations to generate reliable confidence intervals. ED = emergency department; No. = number; Obs. = observations; CT = computed tomography; MRI = magnetic resonance imaging; MSA = Metropolitan Statistical Area.

Summary of demographic, clinical and hospital data for visits with a diagnosis of PE and all other ED visits for comparison.

Approximately 75.1% of patient visits with a diagnosis of PE were hemodynamically stable with no significant difference when compared to all other ED visits (P = 0.96). About 57.7% of weighted visits with a diagnosis of PE involved CT or MRI, compared to 11.6% in all other ED visits (P < 0.0001). During 2005–10, the period where MRI and CT were separate variables, there were no visits with a diagnosis of PE involving MRI alone. MRI was performed in 3 unweighted patient visits for PE. CT was also performed during all these visits.

About 86.3% of patients with a diagnosis of PE were admitted or transferred to another medical facility from the ED, compared to 14.7% of patients seen with all other diagnoses (P < 0.0001). There were 7 unweighted patient visits where the patient was dead on arrival or died in the ED during the study period, which would translate roughly to 3.4% of weighted visits, but the sample is too small to generate robust CIs. From 2005–10, when hospital discharge data were available, 3 unweighted patient visits were admitted and died prior to discharge, representing approximately 2.8% of all ED visits for PE admitted during this period, though the number of observations does not permit generation of robust CIs.

**Figure 1:**
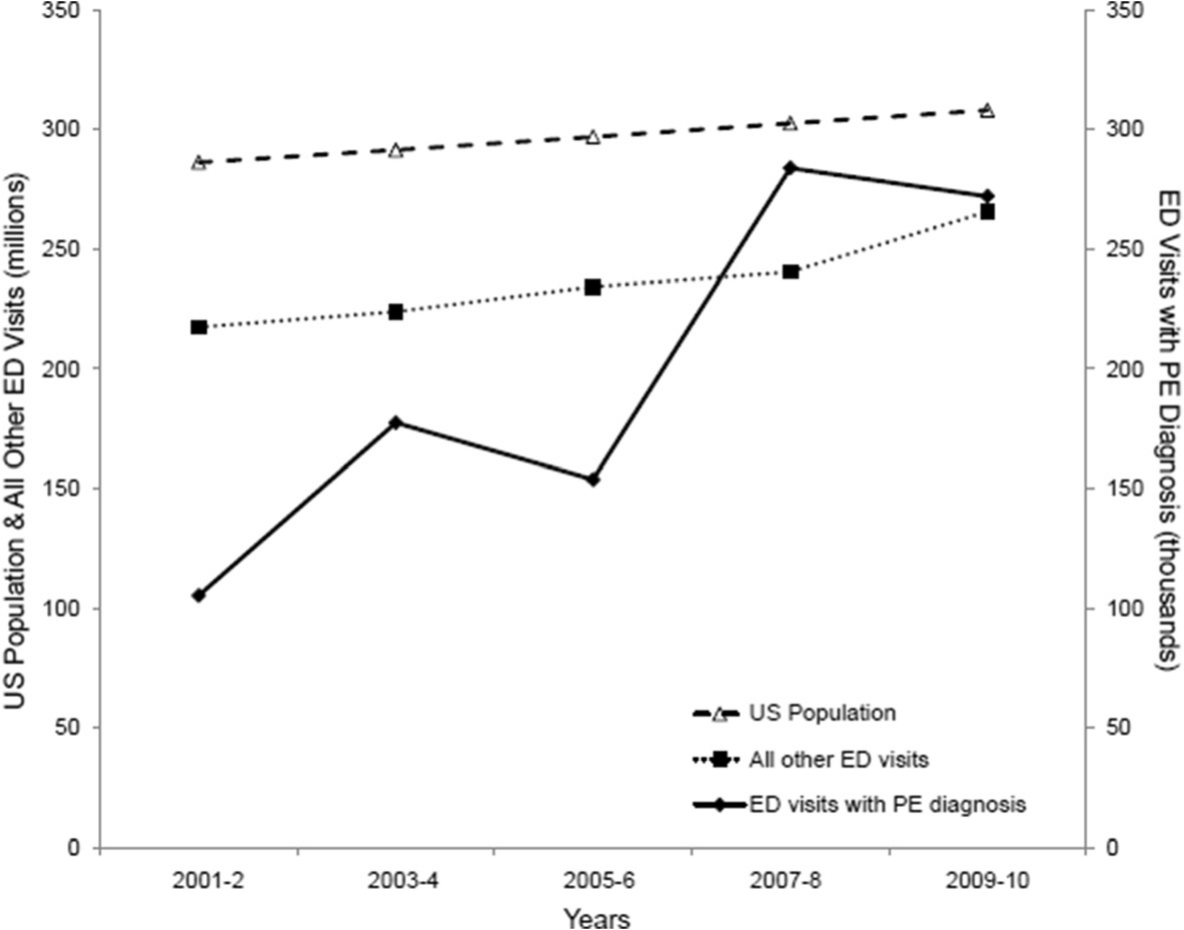
**Trends in ED visits with a diagnosis of PE over time.** Shows trends in US population, ED visits with a diagnosis of PE and all other ED visits over time in two-year intervals.

Over the study period there was a gradual increase in the total US population and number of ED visits. This same period experienced a significant rise in the number of ED visits for PE (see Figure 1). In 2009 and 2010 combined there were 267,000 (95% CI 184,000-351,000) ED visits with a diagnosis of PE compared with 105,000 (95% CI 67,000-143,000) in 2001 and 2002. Among patients seen in the ED, the likelihood of having a diagnosis of PE per year increased significantly over the period 2001 to 2010 (OR 1.091, 95% CI 1.034-1.152, P = 0.002 for trend) when adjusted for demographic (age, race, sex) and hospital information (region, Metropolitan Statistical Area status). In contrast, when further adjusted for the use of CT and/or MRI, the likelihood of having a diagnosis of PE per year did not change over the study period (OR 1.041, 95% CI 0.987-1.097, P = 0.14). Table 2 summarizes the association of demographic variables and hospital information with the likelihood of a visit for PE.

**Table 2:** **Results of logistic regression model assessing relationship between demographic data and likelihood of PE diagnosis**

		**Excluding CT and/or MRI data**				**Including CT and/or MRI data**			
		**OR**	**95%** **CI**		**P-value**	**OR**	**95%** **CI**		**P-value**
Time (in 1 year intervals)		1.091	(1.034	1.152)	0.002	1.041	(0.987	1.097)	0.14
Age		1.038	(1.033	1.042)	<0.001	1.031	(1.026	1.036)	<0.001
Male		1.094	(0.805	1.487)	0.560	1.098	(0.807	1.492)	0.55
Race	*White*	1	--	--	--	1	--	--	--
	*Black*	1.050	(0.728	1.515)	0.790	1.162	(0.803	1.682)	0.43
	*Other*	0.200	(0.074	0.542)	0.002	0.197	(0.074	0.528)	0.001
Region	*Northeast*	1	--	--	--	1	--	--	--
	*Midwest*	1.518	(0.978	2.348)	0.061	1.463	(0.957	2.237)	0.079
	*South*	0.913	(0.547	1.523)	0.730	0.924	(0.562	1.521)	0.76
	*West*	1.511	(0 .967	2.364)	0.070	1.589	(1.019	2.478)	0.041
Within MSA		0.803	(0.466	1.386)	0.430	1.012	(0.591	1.731)	0.97
Use of CT and/or MRI		*Not included*				7.003	(5.100	9.616)	<0.001

Demographic variables and hospital information associated with the likelihood of a PE diagnoses among patients seen in the ED. Table includes data both adjusted and not adjusted for the use of CT and/or MRI.

Table 3 compares the first five years of the study period (2001–5) with the later five years (2006–10). A significantly greater proportion of patients underwent CT or MRI imaging in the later period (69.2% compared to 37.9%, P < 0.0001). There was no difference in sex or race between the two time periods. Although a greater proportion of patients during the later years were 65 years and over (48.0% compared with 37.7%), this difference did not reach statistical significance (P = 0.17). There was no change in the proportion of patients who were hemodynamically unstable or rate of admission/transfer. While not shown in the table, there was also no difference between 5 year periods in geographic locations (geographic region or proportion in a Metropolitan Statistical Area) where PEs occurred (P = 0.65 and P = 0.32, respectively).

**Table 3:** **Comparison of PE ED visits during first and second half of study period**

		**ED visits for PE**						
		**2001-2005**			**2006-2010**			
**Characteristic**		**No. of obs.**	**Weighted %**	**(95%** **CI)**	**No. of obs.**	**Weighted %**	**(95%** **CI)**	**P-value**
Age	Under 65 years	75	62.3%	(5.6%)	87	52.1%	(4.5%)	0.17
	65 years and over	48	37.7%	(5.6%)	73	48.0%	(4.5%)	
Sex	Male	53	43.9%	(5.6%)	70	46.0%	(5.1%)	0.78
	Female	70	56.1%	(5.6%)	90	54.0%	(5.1%)	
Race	White	100	80.1%	(5.8%)	123	80.1%	(4.0%)	0.87
	Black	21	18.8%	**	34	19.3%	(4.0%)	
	Other	2	1.1%	**	3	0.6%	**	
Hemodynamics	Stable	84	75.2%	(4.7%)	119	75.1%	(4.6%)	0.99
	Unstable	30	24.8%	(4.7%)	41	24.9%	(4.6%)	
CT or MRI	Yes	46	37.9%	(5.7%)	108	69.2%	(4.7%)	<0.0001
	No	77	62.1%	(5.7%)	52	30.8%	(4.7%)	
Disposition	Admission/Transfer	96	88.8%	(3.0%)	137	90.5%	(2.8%)	0.59
	Discharge from ED	23	11.8%	**	20	9.5%	**	
	Death on Arrival/in ED	4	1.9%	**	3	4.2%	**	

**Indicates insufficient observations to generate reliable confidence intervals.

Comparison of demographic, clinical and hospital data between the first (2001–5) and second (2006–10) half of the study period associated with ED visits for PE.

## Discussion

Over the past decade, the diagnosis of PE has remained an important public health issue. Our findings here demonstrate that the number of patients seen in the ED who were given a diagnosis of PE more than doubled from 2001 to 2010. However, when adjusted for CT utilization, there was no significant rise over this time period in the likelihood of a diagnosis of PE among patients seen in the ED. Thus, the apparent rise in ED visits with a diagnosis of PE may be attributed in large part to the increased availability and use of CTPA, rather than reflecting a true rise in the incidence of PEs in the US. An aging population may have contributed to a true increase in PE diagnosis [18,19], but our data do not show a significant change in the proportion of patients with a diagnosis of PE 65 years and over during the study period. None of the other variables assessed changed significantly during the study period, including sex, race, geographic region, Metropolitan Statistical Area status, hemodynamic status, and admission to the hospital. Overall then, our data suggest that the use of CTPA has contributed to the substantial increase in PE diagnosis.

Our data further suggest that many patients given a diagnosis of PE in the ED may have been unnecessarily hospitalized. Most patients in EDs with a diagnosis of PE were hemodynamically stable at presentation, with stability defined using cutoff values from the pulmonary embolism severity index for prognostication in patients with acute symptomatic PE [14,15]. Subsequent in-hospital mortality was under 3%, which agrees with the low mortality rates recently reported in in 22 community and academic EDs in the US [10]. The vast majority of patients with a diagnosis of PE were nonetheless admitted to the hospital. Rates of admission for patients with a diagnosis of PE in the ED remained stable throughout the study period at approximately 86%, compared to approximately 15% for all other ED visits.

There is now good evidence that hemodynamically stable patients should be considered candidates for outpatient management. We observed, however, than only about 10% of ED PE patients were discharged from the ED for outpatient management. Recommended prerequisites for outpatient management include the absence of serious co-morbid conditions (e.g. significant heart disease, renal or liver failure) or recent bleeding, as well as adequate social support [20-24]. Recently published data suggest that approximately 50% of patients with acute PE can be treated safely as outpatients [20-22]. There is great potential for healthcare cost savings; the average cost of admission for PE in a recent analysis was over $8,000 [25].

Over the study period, ED visits that included a diagnosis of PE comprised approximately 0.08% of all ED visits. Those diagnosed with PE in the ED were older than the population seen in the ED with diagnoses exclusive of PE, not surprising since older age is a known risk factor for PE [26]. Nonetheless, most visits with a diagnosis of PE in the ED (approximately 56%) were under 65 years old, which agrees with data recently reported in a multicenter study assessing clinical characteristics of suspected or confirmed PEs in the ED [10]. Our data further reinforces the need to consider PE among younger patients.

There were a number of limitations to our study. The retrospective design limits the data available for analysis. For example, the use of ICD-9 codes to identify visits for PE does not differentiate between suspected and confirmed PEs. Furthermore, we cannot assess how the PE was diagnosed and whether or not the PE was acute, chronic, or recurrent. While the use of ICD-9 codes has been validated in hospitalized patients [12], there is data to suggest that PE diagnostic codes reported in EDs should be used with caution [27]. It is therefore possible that the true incidence of PE has not changed and that our observed increase is largely related providers more often including PE as a provisional diagnosis in the ED. We nonetheless feel this is an important finding – ED doctors may be more often considering the diagnosis of PE in the era of more readily available CTPA. It is also possible that patients with chronic or recurrent PEs are presenting to the ED with increased frequency. While it is likely that some PEs were not diagnosed in the ED, we do not believe this underestimation would have changed over time and therefore trend analysis should not have been significantly impacted. Since NHAMCS had a combined CT and MRI variable between 2001 and 2004, we cannot distinguish which of the two technologies were utilized. However, this is unlikely to pose a significant problem—there were no patient visits with a diagnosis of PE in the ED that underwent MRI alone between 2005 and 2010, and therefore it can be assumed that no or very few patients underwent MRI alone when the combined CT/MRI variable was reported positive between 2001 and 2004. NHAMCS does not include data on the utilization of ventilation/perfusion lung scans. Since mortality in patients diagnosed with PE was infrequent, we were unable to generate robust confidence intervals for these characteristics or assess for trends. We can, however, confidently conclude that in-hospital mortality in patients diagnosed with PE in the ED was very low and this agrees with recently reported data [10]. Finally, it is possible that the changing demographics of the US population could account for the increase in proportion of ED visits with a diagnosis of PE.

## Conclusions

Our data demonstrate that the burden of PE diagnosis in US EDs has risen substantially over the past decade. This increase is at least in part attributable to greater CT utilization. The majority of patients with an ED diagnosis of PE were hemodynamically stable and therefore likely candidates for outpatient management, yet over eighty percent were admitted for in hospital treatment. There may be substantial opportunity for cost savings by developing protocols to better define those patients that are appropriate for outpatient management.

## Abbreviations

CDC: Centers for Disease Control and Prevention
CI: Confidence interval
CT: Computed tomography
CTPA: Computed tomography pulmonary angiography
ED: Emergency department
HR: Heart rate
ICD-9: International Classification of Disease, Ninth Revision
MRI: Magnetic resonance imaging
MSA: Metropolitan Statistical Area
NCHS: National Center for Health Statistics
NHAMCS: National Hospital Ambulatory Medical Care Survey
OR: Odds ratio
PE: Pulmonary embolism
SBP: Systolic blood pressure
US: United States
